# Nurse leader agency: Creating an environment conducive to support for graduate nurses

**DOI:** 10.1111/jonm.13561

**Published:** 2022-03-02

**Authors:** Ashlyn Sahay, Eileen Willis, Debra Kerr, Bodil Rasmussen

**Affiliations:** ^1^ School of Nursing, Midwifery and Social Sciences Central Queensland University Mackay Queensland Australia; ^2^ College of Nursing and Health Sciences Flinders University Adelaide Australia; ^3^ School of Nursing and Midwifery, Faculty of Health Deakin University Victoria Australia

**Keywords:** agency, graduate nurses, nurse leaders, patient safety, structuration theory, structure

## Abstract

**Aim:**

The aim of the study was to gain insight on how nurse leaders manage a culture of safety for graduate nurses.

**Background:**

Current theoretical approaches to safety culture tend towards a checklist approach that focuses on institutional characteristics, failing to examine the quality of interpersonal relationships. These interpersonal interactions are often seen as separate from the institutional realities of resource allocation, nurse–patient ratios, patient acuity or throughput. A theoretical approach is required to illuminate the dialectic between the structure of an organisation and the agency created by nurse leaders to promote patient safety.

**Design:**

Qualitative exploratory descriptive study.

**Methods:**

Semi‐structured interviews were undertaken with 24 nurse leaders from hospital and aged care settings. Thematic analysis and Giddens structuration theory was used to describe the findings.

**Results:**

Nurse leaders identified a range of reciprocal communicative and cultural norms and values, decision‐making processes, personal nursing philosophies, strategies and operational procedures to foster patient safety and mentor graduate nurses. The mentoring of graduate nurses included fostering critical thinking, building and affirming formal structural practices such as handover, teamwork, medication protocols and care plans.

**Conclusions:**

The study provides insight into how nurse leaders foster a culture of safety. Emphasis is placed on how agency in nurse leaders creates an environment conducive to learning and support for graduate nurses.

**Implications for Nursing Management:**

Nurse leadership functions and decision‐making capacity hinges on multiple factors including practicing agency and aspects of the social structure such as the rules for safe communication, and the various institutional protocols. Nurse leaders enforce these forms of engagement and practice through their legitimation as leaders. They have both allocative and authoritative resources; they can command resources, direct staff to attend to patients and/or clinical tasks, mentor, guide, assign, correct and encourage with the authority vested in them by the formal structure of the organisation. In doing so, they sustain the structure and reinforce it.

## 1 INTRODUCTION

This paper explores the way in which nurse leaders (NLs) actively pursue patient safety through strategic interactions with graduate nurses (GNs). In particular, NL mentorship at the ward level is important in fostering good work ethics, maintaining staff motivation and overall supportive and collegial workplace habits. In this paper, we present an overview of how the culture in nursing impacts patient safety. We introduce Giddens (1984) theory of structuration as a way of understanding how NLs at the ward level consciously practice creating a culture of safety with GNs. Additionally, we demonstrate how NLs exercise their agency through mentoring GNs and foster a culture of support conducive to patient safety.

## 2 LITERATURE REVIEW

### 2.1 Nurse role in patient safety management

Nurses are viewed as the ‘safety nets’, overseeing various practices of patient care and related safety practices. They engage in prolonged periods of direct patient care and are considered pivotal to preventing errors (Rosén et al., 2017). Nonetheless, it is widely acknowledged that safe nursing practice can be challenged by inappropriate staffing levels (Twigg et al., 2015), increased patient load, complexity of care and constrained timeframes (Duffield et al., 2011). According to Johnstone et al. (2008), the multi‐faceted nursing role predisposes nurses to making preventable errors, thereby threatening quality of care and overall patient safety (Johnstone et al., 2008). In particular, GNs are at higher risk and vulnerable to errors due to their lack of experience in managing competing priorities and the complex workload. Documented evidence of the challenges faced by GNs includes the ability to handle an intense work environment (Regan et al., 2017), utilization of advanced medical technology (Orbaek et al., 2015) and management of high patient acuity (Duclos‐Miller, 2011).

Of concern, GNs have been found to be largely uncomfortable in approaching senior nurse colleagues for support (Sahay & Willis, 2021). GNs have identified their need for support through effective mentorship (Laschinger et al., 2010) and rely on more experienced nursing colleagues for guidance (Sahay et al., 2015). Nevertheless, some studies indicate that GNs do not seek support from other nurses if the colleague's behaviour is not conducive to learning (Laschinger et al., 2010; Sahay & Willis, 2021). This is where the NL role becomes paramount in fostering a culture of support and collaboration.

### 2.2 Influence of unit‐level nurse leadership on patient safety

Leaders proficient in effectively implementing a culture of safety are known to create a context where safety concerns are constantly discussed. Leader commitment and engagement in safety actions reinforces nurses' adherence to safety protocols while heightening an overall sense of safety for patients, colleagues and self. There is also evidence that NL commitment towards safety initiatives increases reporting of errors and incidents by nurses, thereby, increasing opportunities to learn and develop ‘error wisdom’, which refers to knowledge gained from errors (Reason, 2004, p. ii32). This aligns with the clinical governance framework, which suggests that NLs who foster an environment of support and a ‘just culture’, enable the delivery of safe clinical care. To gain an understanding of the underpinning principles behind a NL's actions, we have adopted Giddens's (1984) structuration theory as it allows exploration between the individual NL's decision making, the culture created and the structure of the organisation.

### 2.3 Structure within the structuration theory

The evaluation of the many organisational changes in hospitals and health services attest to the gap between functionalist notions of structure and the ethnomethodological realities of everyday interactions. A social system exists within these structures as it is reproduced over time through the practices of its agents/individuals and groups (Mustafa & Mische, 1998). Giddens describes the ‘social system as having three dimensions: signification, domination and legitimation, that in turn reflect three forms of interaction: communication, exercise of power and sanction’ (Whittington, 2015, p. 148). Signification denotes the rules governing communication, and legitimation, the formal and informal rules and legal requirements of interaction. Domination depends on both allocative and authoritative resources. Allocative resource capacity refers to objects and materials, while authoritative denotes command over individuals. Both allocative and authoritative resources are reproduced through agency: the first in practical ways through material production and the second within the social space where humans come together in actions of production, and service. It is within the authoritative realm that humans form groups and associations, and also where their position influences their life chances (Giddens, 1984). These forms of interactions and dimensions are not mutually exclusive; for example, the legitimation of the position of nurse manager speaks to their power and to the forms of communication they engage in, including the significance of their discourse (Whittington, 2015).

In Giddens's framework, structure is the various practices, behaviours, rules and norms that individuals operate under. It does include institutions such as the family, the legal or education system, but Giddens focuses more on the rules and patterns of everyday life (virtual) that create social structures, than the institutions themselves that provide the framework for behaviour (Braithwaite, 2006). Giddens proposes that structure and agency are co‐dependent; that in effect the practices of the agent create the structure. One does not exist without the other. It is the agency of the various individuals that creates structure as they act upon the world. The structure of a society and its agents are in interaction with each other; they only exist because of the other, although they also exist independently. Individuals can think, reflect and act independently of a social structure, but what they think and do is a reflection in one way or another of the mutual dependence on the structure.

### 2.4 Agency and structuration theory

The important starting point for understanding agency is in its reflexive capacity. Giddens refers to the intentional and purposeful direction of human behaviour as constituting agency. He distinguishes this act of agency from unintentional acts, arguing that agency is practiced when the actor is fully conscious, has a sense of what the outcome will be and that it is not a spontaneous or habitual action (Eteläpelto et al., 2013).

**FIGURE 1:**
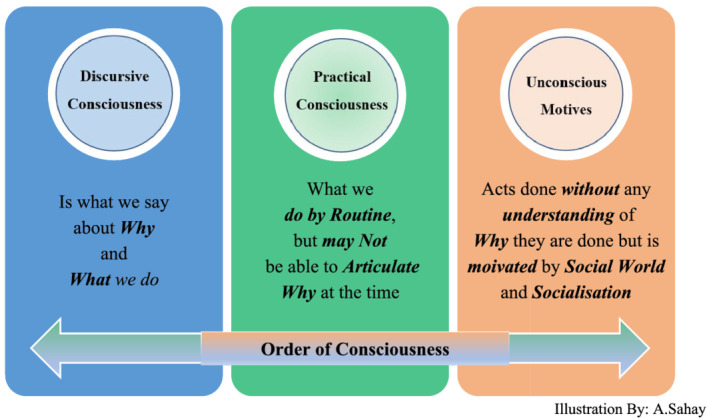
Order of consciousness. Illustration by A. Sahay

In order to elaborate on this purposeful action of the agent, Giddens makes a distinction between the concepts of discursive consciousness; practical consciousness; and unconscious motives/cognition (Giddens, 1984; Mustafa & Mische, 1998). These can be defined in order of consciousness: ***discursive consciousness*** is what we say about why and what we do; ***practical consciousness*** is what we do, by routine, even if we are not necessarily able to articulate why at the time; while ***unconscious motives*** are acts done without immediate understanding of why they are done, but clearly motivated by aspects of our social world and socialization. This is outlined in Figure 1.

Reflexivity operates mainly at the level of discursive and practical consciousness and is often articulated in hindsight (Braithwaite, 2006) or based on experience or practical evaluation of past situations. It is the deliberate decision making about the appropriate course of action, even when the individual knows the evidence to be flimsy (Mustafa & Mische, 1998), or within the framework of trust based on ontological security (Giddens, 1990).

Giddens identifies that agency operates at the level of practical consciousness, as it is a cognitive exercise that leads us to reflect on why we do what we do, to think it through. These discursive and practical conscious acts are bound by the structures within which we operate. Our reflections follow the cultural and social rules of our particular time and place, which can be loosely defined as culture. The structure is bound by a particular time and space, given it is constituted by agents acting reflexivity. As Giddens notes, ‘All human action is carried on by knowledgeable agents who both construct the social world through their action, but yet whose action is also conditioned and constrained by the very world of their creation’ (Giddens, 1981, p. 54). In arguing for intentional action, Giddens is clear that the individual must have the power both in terms of authority and resources to bring about the action; intention or knowledge of typification or schema is not agency. It requires action and an awareness that this is what we are doing, even when done under duress (Eteläpelto et al., 2013).

### 2.5 Culture, structure and agency

We can further understand the duality of agency and structure by reflecting on the place of culture within the framework. The practices performed by the agents‐individuals constitute the given culture of an organisation. The patterned form of interactions is of cultural practice. It includes the way we talk, act, or what is espoused. Braithwaite notes organisational culture is ‘the way things are done around here’ (page 97). In order for society to operate and function, there is a shared understanding of many of the practices, beliefs and norms. It is not simply a duality between one individual acting upon the social that creates the structure, but many individuals with a shared understanding of the norms, values and practices that create the structural component of Giddens's agency structure including the culture.

### 2.6 Agency and identity

The interpretation the individual agent assigns to these cultural norms, values, symbols, beliefs and practices and how they respond to them builds up and reflects their identity. Without it there would be no redemption, personal change or self‐improvement. It is our self‐concept or identity that shapes our intentional actions, but they in turn are influenced in concert with social change. How individual NLs support their team and mentor GNs speaks to their agency, the culture they create on the ward and their own sense of identity as a leader.

## 3 METHODS

Data presented here is from a larger study. The aim of the study was to gain insight on how NLs managed a culture of safety for GNs. Ethics approval was sought from a large hospital and health service and an academic institution. Data were gathered by the first author through a series of interviews with 24 NLs in Australia. The NLs were purposively selected and interviewed in a venue of their choice between May 2016 and June 2017. They were invited to engage in the study through responding to advertisements posted across several hospitals in Victoria (Australia) and in local libraries, and/or snowball approach. The interview schedule asked NLs to reflect on how they supported GNs to provide safe and quality patient care. The interviews were collected till data saturation, with member checking occurring with the NLs through a process of identifying themes. Table 1 provides the demographic details of the NLs.

**TABLE 1:** Summary of nurse leader demographic details

Demographic details	Number (*N* = 24)	Percentage (%)
***Gender***		
Female	21	87.5
Male	3	12.5
***Interview mode***		
Face to face	20	83.3
Telephone	4	16.7
***Employment capacity***		
Full‐time	11	45.8
Part‐time	13	54.2
***Years in leadership role***		
1–10	9	37.5
11–20	6	25
21–30	6	25
31–40	3	12.5
***Leadership positions held***		
Nurse unit manager	5	20.8
Associate nurse unit manager	4	16.7
Nurse educator	4	16.7
Team leader	4	16.7
Clinical nurse specialist	3	12.5
Aged Care facility manager	2	8.3
Hospital nurse manager	2	8.3
***Areas worked***		
Aged Care	7	29.2
Intensive Care Unit (Adult and Neonatal)	5	20.8
Acute Medical or Surgical Ward	4	16.7
Primary Health care	4	16.7
Emergency Department	2	8.3
Peadiatrics	2	8. 3

### 3.1 Analysis

The methods used in this paper are limited to an analysis of what these NLs said they did, rather than to what their practice was. In taking this approach, we have examined the interviews and used Braun and Clarke's (2012) six step guide to thematically draw on the concept of agency and its duality with structure. This has allowed us to take note of the words used, the norms espoused and the practices sanctioned. The interviews were examined to answer three questions about the NL's agency. These were as follows:

1. What did NLs intentionally do to assist GNs to ensure patient safety and/or to develop their critical thinking to enhance patient safety?
2. How were these actions linked to official practices to ensure patient safety?
3. What do these practices say about the safety culture of NLs at the ward‐level in their interactions with GNs?

The process of analysis involved reading the interviews with the three questions in mind for relevant responses and surrounding textual material. This was done in two stages with the first author organising the interview quotes into generalized themes, and the second author linked them to the research questions noted above. This approach mirrors template analysis where the researcher reads the data with a specific theoretical framework in mind and searches the text for confirmation (Waring & Wainwright, 2008). The pathway does not exhaust the data, or necessarily capture all there is to say about the context. Rather it identifies one of the salient features within the data and illustrates the theoretical links (King, 2004). The themes identified by the second author were then checked by the first author for coherence with the literature and the original thematic analysis. The two themes reflecting NL's agency included: Assisting GNs to maintain patient safety (through enhancing critical thinking) and enhancing structure through sanctioning official practices (through teamwork and handover).

## 4 FINDINGS

### 4.1 Theme one: Assisting GNs to maintain patient safety

This theme focuses primarily on how NLs foster critical thinking.

### 4.2 Supporting GNs to enhance critical thinking

This sub‐theme refers to NL perceptions on how nurse‐to‐nurse interactions strengthens GN's critical thinking skills. NLs expressed the view that supportive interactions advance GNs' ability to make appropriate clinical judgements and to apply sound reasoning to provide safe and high‐quality patient care. Nurses are expected to assess and evaluate a situation from multiple perspectives, develop rationalized care plans and implement nursing practice that maintains patient safety. NLs stressed the importance of understanding the context, as well as the underpinning reasoning to specific nurse actions rather than being task centred. The comment below points to habitual action, or action without agency:

*I think some people [GNs] … just do things, and it's kind of like ‘why would you do that?’ and they do not have a reason. They are just jobs [clinical tasks] they do every half‐hour. The problem seems deeper than that, and I think it's more than reflective practice. They're just doing, rather than thinking, about what they are doing … you need to critically think and apply it to the care you are giving … asking why … people continue doing things without linking the two* (NL 15).

To help develop GNs' critical thinking, NLs employed various approaches to increase safe practice.

*A [GN] felt that the patient's stomach pain had increased over the last 24 hours and wanted some advice. I told him to think about the basics and eliminate using a process of elimination and think critically. Had the patient's bowels moved? Let us do bladder scan to make sure the pain is not related to retention, something that could be easily rectified. After doing that, then we escalated to the doctor to obtain pain relief for the patient* (NL 5).

Quality of feedback was reported as essential to ensure GNs developed critical thinking skills and was seen to build confidence and reinforce safe practice. However, the way in which feedback is delivered by experienced nurses affects GNs' confidence. NL 11 explained that feedback is important in building the GNs' confidence. In her view, the way in which feedback is delivered affects how GNs perceive nursing:

*I think very new nurses [GNs] … get a lot from feedback and perhaps sometimes even if I am biting my tongue [not saying anything] and thinking, ‘You could do that quicker’, I would not necessarily say so. A ‘pat on the back’ [praise] goes a long way and can definitely affect how they feel about nursing and what sort of a nurse they will turn out to be if they are exposed to positive reinforcement* (NL 11).

Other NLs commented that some experienced nurses may not have the proficiency to deliver constructive feedback. In her view, nurses would rather discuss performance issues with other NLs, avoiding an interaction with the actual individual to discuss how their clinical performance could be improved:

*It's about being upfront and honest with GNs and being able to give constructive feedback. I do not think nurses are very good at giving constructive feedback. They say, ‘A graduate is not doing this, this or this’. And then I say, ‘Well have you told her?’ And they say, ‘no because she might get offended’. I think she will get more offended if she finds out you are whining about it to me. How about saying, “The way you are doing this is not great, why not try and do this, this way. It's going to impact on the patient, so you should really be doing it this way” (* *NL* 15).

Nonetheless, other NLs expressed the view that validating GNs' concerns was an opportunity to deliver constructive feedback. The NLs commented that GNs lack confidence to inform effective clinical judgements and therefore rely on experienced nurses to validate their plan of care. NL 8 recalled that when GNs seek validation, she would either validate concerns and/or offer constructive feedback.

*Being in a leadership role, it's important you listen to them [GNs]. You validate that they should be concerned, ‘You've every right to call the medical officer,’ ‘Do not be fearful,’ … once they hear you say this, they will think ‘Yeah, something needs to be done’. Or you might say, ‘Have you done this assessment? Maybe do that prior to ringing.’ So, it's taking the time, debriefing with them, and then offering them either some advice or some validation* (NL 8).

Feedback can be given in various ways, and be viewed as positive, constructive and/or negative. However, the way feedback is perceived or interpreted could also play a role in how it is processed. The data indicate the quality of feedback impacts on GN confidence, competence and patient safety outcomes.

### 4.3 Theme two: Enhancing structure through sanctioning official practices

As Giddens (1984) argues the decisions or agency of the individual finds expression in practice. The decisions made by NLs must be made concrete at the ward level in how handover or the medication rounds are conducted, and what pathways and protocols are followed. The discussion below reflects on how NLs created teams and how they used the structure of handover to make concrete the norms and values they wished to enforce in the interest of patient safety. Therefore, answering the question: How were these actions linked to structure and culture?

### 4.4 Working as a team to improve patient care

NLs explained that interactions improve when nurses accepted each other as equals and there is clarity about team member roles:

*[Teamwork is] about engaging with each other and seeing each other as equals and understanding that we all have a role to play … If we lack that cohesiveness and collaboration, then it impacts on patient outcomes* (NL 12).

However, NLs recognized that working as a team can sometimes present challenges, due to the multiple personalities that make‐up a team. They noted that some nurses work well together, while others struggle to collaborate and interact with one another:

*When nurses are unable to settle their differences, then it makes people work in isolation, and you cannot do that. Nurses do not discuss and clarify things with one another, and that's when mistakes happen. You need to work as a team and stay professional even if you reserve less favorable thoughts for your team members* (NL 1).

Another NL emphasized the importance of appropriately matching and/or pairing team members to increase collegiality and team efficiency.

*If I put three disinterested people in a pod, the care level would be 50%; the documentation would not be up to scratch … the interactions will be poor. Pairing them with a stronger team member I find will get them to step‐up a level … You tend to find that's when a team comes together. It's being able to identify where to place someone in the unit so that it is a cohesive group* (NL 18).

NLs also referred to constant interruptions during verbal handovers as another factor that affects the quality of nurse‐to‐nurse interactions. This subsequently impacts on the quality and type of information transferred, thereby resulting in omission of vital patient information. The data revealed that interruptions during handover were caused by two factors: environmental factors (e.g., patient discharges, phone calls, family concerns and new patient admissions,); and incoming nurse receiving the handover.

NLs reported that interruptions influenced by the ward environment were detrimental to the complete transfer of patient care information. This consequently resulted in the omission of information, such as planning to undertake blood tests and administration of antibiotics and other medications:

*When the handover time is interrupted from the environment, then certain things do not get handed over properly. For example, blood tests do not get done because it's not told or not written down in the care plan. Antibiotics are not given when they are not pointed out, perhaps the [incoming] nurse has not got back to looking at the medication chart until two hours later and then realises that she's missed giving a medication – an antibiotic and that impacts on the quality of care* (NL 8).

Further, some NLs commented that interruptions made by the incoming nurse during the handover process also impacted on the quality of information transferred. In one NL's view, constant interruptions by incoming nurses during handover are distracting and disrupts thought processes of the outgoing nurse delivering the handover. This subsequently results in the omission of vital patient information. NL 14 explained:

*You are trying to follow the ISBAR, but you have got a nurse [who] constantly interrupts … leads to omission of some information … You are distracted, and thoughts are interrupted … which then leads to more omissions* (NL 14).

## 5 DISCUSSION

As the first theme demonstrates, NLs actively mentor GNs through a process of ensuring they develop habits of reflective practice. They model this through a careful process of mentoring, modelling and questioning GNs about their patient care. This is an open act of discursive consciousness on their part, but it is also an attempt to raise the consciousness/awareness of the GN. Many of the quotes demonstrate this process of leading the GN to a process of critical thinking—a shorthand for agency. The NLs provided advice in a structured way and monitor the feedback they provide to GNs to ensure that it builds their confidence, rather than increasing their stress levels. At times they bracketed out other deficits such as time management to build the GN's confidence. They are mindful of the pitfalls of routinization and practice as habit, rather than active agency, validate the decisions made by GNs and encourage other senior staff to be frank, but positive in their interactions.

Similarly, this reflective agency is made concrete in the structures of practice within the ward. Teamwork is encouraged and organised around skill, motivation and expertise. A culture of sharing the load is encouraged as it builds alliances and safeguards against errors. The NLs are mindful that if colleagues do not get along with each other this is detrimental to patient safety. Structural practices are rigorously enforced to ensure that the aims of the institution are met. The example provided here is of handover. Several NLs stressed the importance of communication using the ISBAR format: *I*dentifying and *S*olving *BAR*riers to effective clinical handover (Hunter New England, 2009). It represents a set process or structure for handover instigated to mitigate errors. They also stressed the importance of maintaining medication charts, patient care plans, protocols and procedures, and minimizing interruptions. Interruptions during handover or claims to being too busy to communicate adequately were not tolerated. All these practices go towards creating the structure and culture on the ward, hospital and wider organisation with its focus on patient safety and are part of the legitimate exercise of power accorded to NLs.

The NLs also had the power to allocate staff to particular areas indicating their command over the labour of individual staff. They signalled to the team the need to be approachable for junior nursing staff, however, were aware that not all senior nurses were accommodating. What we did not find in the data, were examples of the approaches they took to change behaviours for senior staff who did not act as mentors for GNs. This suggests that their agency was not always positive.

A careful reading of the NL comments also provides an analysis of the culture of safety required on any ward where there are GNs. These NLs, exhibit discursive consciousness in what they say they do, and how they build up the culture of the ward or unit, in how they mentor GNs, encourage teamwork, or guard against routine habituated practice (Mustafa & Mische, 1998). In many ways much of this will have become practical consciousness. They may not be able to identify why they do it at the time, but spontaneously act in the interest of the structure of the organisation.

### 5.1 Limitation

The study was designed to be descriptive. The participants were largely from within one state which makes the study geographically limited. However, participants were from different specialty areas, which enabled the collection of data from a diverse range of nursing settings. Furthermore, as the study aim was examined from the perspective of NLs only, it is not representative of all nurse groups (i.e., nurses at various levels of work experience) or nursing roles. It is possible that perceptions of the impact of nurse‐to‐nurse interactions on patient safety outcomes may differ among other nurse groups. Despite these limitations, the study has relevance, and important implications for practice and future research initiatives.

## 6 CONCLUSION

In this article we have outlined the agency of NLs as they manage mentoring of GNs in order to ensure they develop the necessary critical skills, clinical expertise and time management. These skills are required to ensure patient safety. We demonstrated the way in which these NLs consciously instigate lines of communication with GNs as part of their mentoring. We also demonstrated the way they use their legitimate authority to ensure the various structural practices, such as handover, are adhered too. The very process of interviewing these NLs allowed them to bring to the surface (discursive consciousness) an awareness of how they create and re‐create a culture of safety within the ward, while simultaneously mentoring GNs. We also highlighted their awareness of practical consciousness acts which are routine and lie somewhere between consciousness and the habitual. We have suggested that unconscious actions, while not always accorded agency, do arise from the individual's orientation or socialization and for that reason have currency. Importantly, the paper points to the fact that patient safety goes beyond the number of staff allocated to a shift, or the material resources available. It extends to the very culture of a ward or hospital, and to the interactions between nurses, doctors and other health staff. How these interactions play out, how they contribute to the expertise of junior staff are all matters of patient safety.

### 6.1 Implications for nursing management

Nurse leadership functions and decision‐making capacity hinges on multiple factors including practicing agency and aspects of the social structure such as the rules for safe communication, and the various institutional protocols. NLs enforce these forms of engagement and practice through their legitimation as leaders. They have both allocative and authoritative resources; they can command resources, direct staff to attend to patients and/or clinical tasks, mentor, guide, assign, correct and encourage with the authority vested in them by the formal structure of the organisation. In doing so, they sustain the structure and reinforce it.

## CONFLICT OF INTEREST

None.

## AUTHOR'S CONTRIBUTIONS

Interviews, initial thematic analysis, tables and figures were conducted and developed by AS and supervised by DK and BR. Theoretical discussion on structuration theory developed by EW. Final editing by AS and EW.

## ETHICS STATEMENT

Ethics approval for this study was sought and approved from Barwon Health Human Research Ethics Committee (HREC) (approval reference number: 16/30) and Deakin University HREC (DUHREC; approval reference: HEAG‐H28_2016).

## Data Availability

The data that support the findings of this study are available from the corresponding author upon reasonable request.
